# Finding the needle in a haystack: single-balloon enteroscopy to diagnose diffuse large B-cell lymphoma

**DOI:** 10.1055/a-2277-0672

**Published:** 2024-04-09

**Authors:** Reid D. Wasserman, William F. Abel, David Lebel, Klaus Mönkemüller, Paul Yeaton, Vivek Kesar, Varun Kesar

**Affiliations:** 1 246010 Internal Medicine, Virginia Polytechnic Institute and State University Carilion School of Medicine, Roanoke, United States; 2 246010 Pathology, Virginia Polytechnic Institute and State University Carilion School of Medicine, Roanoke, United States; 3 246010 Gastroenterology and Hepatology, Virginia Polytechnic Institute and State University Carilion School of Medicine, Roanoke, United States

A 74-year-old woman who was on warfarin for a past medical history of atrial fibrillation and a mechanical mitral valve was admitted with symptomatic anemia. The patient reported fatigue, shortness of breath, and epigastric abdominal pain, with associated black stools for a week. Her physical examination was unremarkable. Laboratory investigations were notable for a normocytic anemia (hemoglobin 6.2 g/dL), with an international normalized ratio (INR) of 1.6 and a prothrombin time of 19.5 seconds. The patient underwent esophagogastroduodenoscopy and colonoscopy, with there being no evidence of active bleeding. She continued to have persistent drops in her hemoglobin requiring several transfusions during her admission. Subsequent video capsule endoscopy revealed blood intermittently in the mid-to-distal small bowel ( Video 1 ). Computed tomography with enterography of the abdomen and pelvis revealed no acute intra-abdominal process. Push enteroscopy was subsequently performed, with examination of the jejunum being normal ( Fig. 1 ). Further evaluation with single-balloon push enteroscopy revealed an ulcerated lesion of 1 cm, with no active bleeding in the jejunum ( Fig. 2 ). The lesion was biopsied, and the rest of the examination was unremarkable. Biopsies showed an infiltrate of large atypical lymphocytes within the lamina propria of the small bowel with architectural destruction and lymphoepithelial lesions ( Fig. 3 **a** ). PAX-5 immunohistochemical staining identified these atypical lymphocytes to be B cells ( Fig. 3 **b** ). The histology and remaining immunohistochemical stains (not shown) were diagnostic of a diffuse large B-cell lymphoma (DLBCL), activated B-cell subtype.

**Fig. 1:**
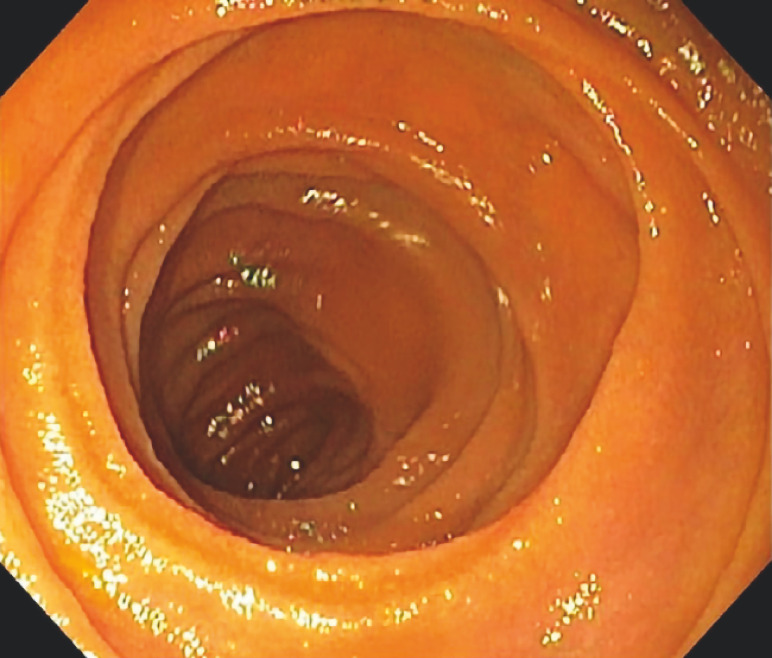
Image during push enteroscopy with normal findings in the jejunum.

**Fig. 2:**
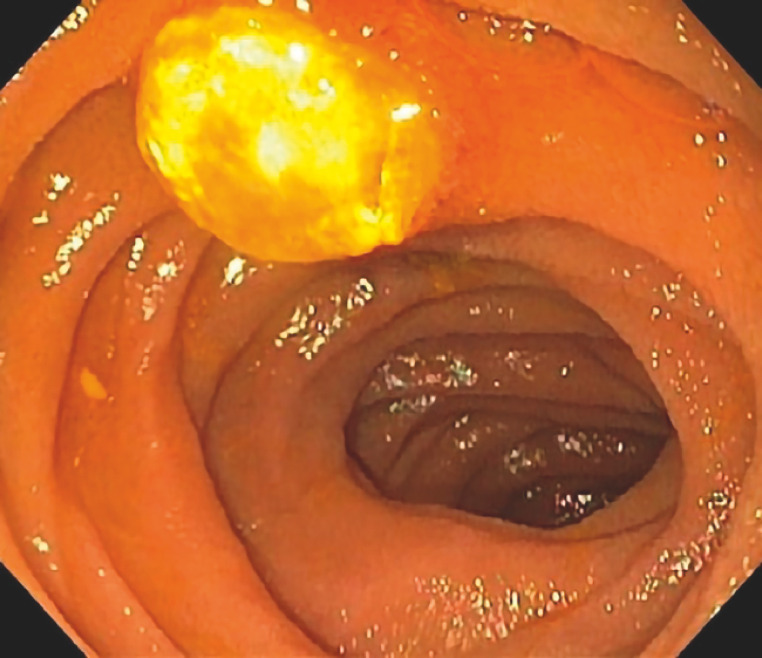
Image during single-balloon push enteroscopy showing a lesion in the jejunum with no evidence of active bleeding.

**Fig. 3:**
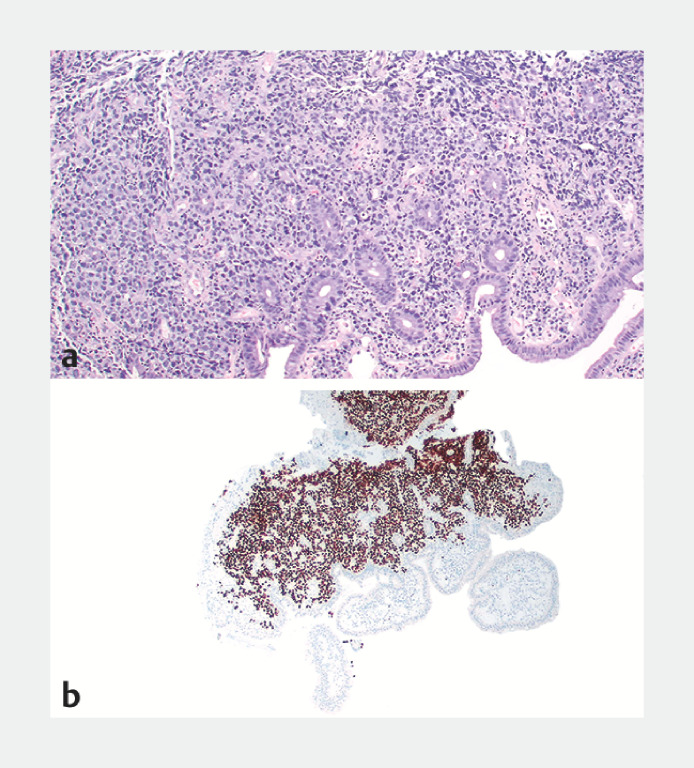
Microscopic appearance of the endoscopic biopsy of the jejunal lesion showing: **a** on hematoxylin and eosin (H&E) staining, an infiltrate of large atypical lymphocytes with architectural distortion and lymphoepithelial lesions; **b** on PAX-5 immunohistochemical staining, positivity of the large atypical lymphocytes, indicating they were B cells.

Jejunal diffuse large B-cell lymphoma is diagnosed using single-balloon enteroscopy.Video 1

DLBCL is the most common type of non-Hodgkin lymphoma 1 . Its presentation may be occult or overt with palpable lymphadenopathy. It may also present at extranodal sites, including the gastrointestinal tract. The stomach and small bowel are the most affected organs. Symptoms of primary gastrointestinal DLBCL include abdominal pain, bowel obstruction, change in bowel habit, or bleeding 2 . Gastrointestinal bleeding can occur during chemotherapy with an incidence up to 11% 2 ; however, there are very few reports in the literature of gastrointestinal bleeding as the presenting symptom of DLBCL.

Endoscopy_UCTN_Code_CCL_1AC_2AC

## Notes

Endoscopy E-Videos https://eref.thieme.de/e-videos
*E-Videos* is an open access online section of the journal *Endoscopy* , reporting on interesting cases and new techniques in gastroenterological endoscopy. All papers include a high-quality video and are published with a Creative Commons CC-BY license. Endoscopy E-Videos qualify for HINARI discounts and waivers and eligibility is automatically checked during the submission process. We grant 100% waivers to articles whose corresponding authors are based in Group A countries and 50% waivers to those who are based in Group B countries as classified by Research4Life (see: https://www.research4life.org/access/eligibility/ ). This section has its own submission website at https://mc.manuscriptcentral.com/e-videos .
